# A Spatio-Temporal Pattern and Socio-Economic Factors Analysis of Improved Sanitation in China, 2006–2015

**DOI:** 10.3390/ijerph15112510

**Published:** 2018-11-09

**Authors:** Qing Luo, Mengjie Zhang, Wei Yao, Yanfen Fu, Haichun Wei, Yong Tao, Jianjun Liu, Hongyan Yao

**Affiliations:** 1 Chinese Center for Disease Control and Prevention, Beijing 102206, China; luoqing@ncrwstg.chinacdc.cn (Q.L.); liujj@chinacdc.cn (J.L.); 2 National Center for Rural Water Supply Technical Guidance, Chinese Center for Disease Control and Prevention, Beijing 102200, China; 18810285279@126.com (M.Z.); yaowei@ncrwstg.chinacdc.cn (W.Y.); fuyf@ncrwstg.chinacdc.cn (Y.F.); weihc@ncrwstg.chinacdc.cn (H.W.); taoyong1958@163.com (Y.T.)

**Keywords:** sanitation, spatial analysis, spatial panel model, rural China

## Abstract

Ensuring an adequate and safe access to sanitation is essential to prevent diseases. Using provincial spatial panel data reported in the China Health Statistical Yearbook and the China Statistical Yearbook, this paper analyzed the spatio-temporal characteristics of improved rural sanitation in 30 Chinese provinces during the period 2006–2015, and analyzed factors that may affect improved sanitation rates in rural China. Spatial autocorrelations of improved sanitation rates were computed via Global and Local Moran’s I firstly, and then, inter-provincial disparities of improved sanitation were assessed by using the Theil index estimator; finally, the spatial panel model was employed to examine the potential socio-economic factors. Spatial autocorrelations results suggested that the provincial improved sanitation rates changes affect both the provinces themselves and the adjacent regions; Analysis of the spatial panel model revealed that factors such as GDP per capita, investment proportion ratio, centralized water supply, rural residents’ expenditure were positively associated with improved sanitation rates, and illiteracy rate of people older than 15 was negatively related with improved sanitation rates. Socio-economic factors had affected the improved sanitation rates in 30 provinces in rural China. Thus, a series of policies, socio-economic measures and personal latrine literacy education should be given to improve the status of improved sanitation rates in rural China.

## 1. Introduction

One of the world’s most urgent issues is a lack of safe water and sanitation. A total of 842,000 deaths from diarrheal diseases each year are thought to be related to water, sanitation and hygiene conditions [1]. Holistic improvements in water and sanitation play a key role in meeting the development goals, reducing child mortality, and improving health in a sustainable way [2]. Huge gaps between the poorest and richest areas, as well as between households, in the categories of water and sanitation were identified in the Millennium Development Goals (MDGs) Report 2015 [3].

Due to the support of the Chinese governments at all levels and the active participation of rural residents [4], China’s sanitation rate has improved significantly from 2000 to 2015. The Joint Monitoring Program report (JMP) of UNICEF and WHO claimed that China had achieved the water and sanitation goals as outlined in the MDGs report by 2015 [5]. However, it is widely demonstrated that low and middle-income countries face undocumented inequalities and environmental health challenges [6]. Moreover, the 2030 Sustainable Development Goals (SDGs) call for the “availability and sustainable management of water and sanitation for all” [7,8]. The historical turning point and development stages in China are outlined below [9].

The first background survey of rural improved sanitation and excrement treatment in rural areas was conducted in 1993. In 1995, the National Patriotic Health Campaign Committee (NPHCC) requested the establishment of a national statistical annual report system for rural sanitation. The report became the statutory statistical work content of the National Bureau of Statistics after 2001. The China Primary Health Care Development Guidelines (2001–2010) and China’s Women Development Program (CWDP) (2001–2010) proposed that the improved sanitation rate (ISR) should reach 65%. The 2002 “Decision of the CPC Central Committee and the State Council on further strengthening rural sanitation work” set a target for the ISR according to different situations in different provinces. After this, the office of NPHCC created the central government’s transfer payment from the Rural Latrines Improvement Project from 2004 to 2008 for the first time. Later, the major public health services focused on improvement in sanitation from 2009 to 2014 (every 3 years). Meanwhile, the national clean and tidy urban and rural environmental sanitation campaign during 2010–2012 and CWDP (2011–2020) set goals for the improved sanitation rate to reach 85%. Thus, it is urgent to obtain a deeper and better understanding of the distribution and evolution of the ISRs, and the influencing factors to improve the ISRs among different provinces. 

Influencing factors for ISR are very complex. Many studies that examined the influencing factors for sanitation rates have been carried out [10,11,12]. They suggested that social development, government commitment and investment, institutional design, social customs, individual hygienic habits economic state, education status, nationality, an individual’s prestige and geographic conditions are the factors that can sometimes influence improved sanitation [13,14]. 

In China, ISR was most affected by economic conditions, geographic disparities and hygienic awareness of individuals in different regions [15,16]. At the same time, imbalanced economic development and individual need for sanitation can contribute to limited sanitation in the western regions, which constrains environmental benefits and weakens the establishment of sustainable economic development [17,18,19]. 

However, the above research results of influencing factors for ISRs were mainly analyzed by the traditional multivariate linear regression method. In view of economic change, climate and geographic conditions, there may be a spatial effect of ISRs among different provinces. Some researchers suggested that the importance of spatial effects in the environmental-related indicators could not be ignored and the use of spatial measurement in the environmental sciences is necessary [20]. Anselin theory held the view that almost all spatial data have the characteristics of spatial dependence or spatial autocorrelation, and the existence of spatial dependence breaks the basic assumption of independence in most classical statistical and econometric analysis [21]. To avoid the deviation in the estimation results caused by the neglect of spatial effects analyzed by the traditional multivariate linear regression models, we choose the spatial econometric model to investigate the factors influencing ISRs. 

In recent years, more and more public health researchers have begun to apply the spatial analysis methods to investigate the influencing factors of diseases related with environmental health and environmental pollution [22,23], it was a trend to merge different spatial effect socio-economic indicators into the panel data model for analyzing the influencing factors of ISRs. However, there is a lack of research that has scientifically analyzed the spatial pattern and spatiotemporal characteristics of ISR, and possible factors that influencing ISRs over time. 

In this paper, our aim is to explore the spatial–temporal distribution of ISRs in 30 provinces in rural China from 2006 to 2015, and examine the possible socio-economic factors (centralized water supply, GDP per capita, urbanization, rural residents’ expenditure, illiteracy rate of people older than 15, etc.) of ISRs by spatial econometric models. The results will provide basis for future policy and measures to improve current status of ISRs in rural China. 

## 2. Materials and Methods

### 2.1. Data Sources

Our data is mainly acquired from the China Health Statistical Yearbook and the China Statistical Yearbook (CSY) during the period 2006–2015. CHSY and CSY are published national reports that cover 31 provinces, municipalities and autonomous regions of China except Hong Kong, Macao and Taiwan (hereafter referred to as 31 provinces). This information is updated each year, with data being collected from more than 2400 counties in 31 provinces.

Data sources and expected results are shown in Table 1.

This is a secondary analysis of the previously published and collected survey data; thus, ethical approval was not required for this work.

### 2.2. Definition of the Indicators

New sanitation refers to latrines with clean walls and a roof, with no leakage in the pit of the latrine and no flies. These include a flushing toilet, piped sewer system, septic tank, a ventilated improved pit latrine (VIP), a pit latrine with slab, etc. 

The investment proportion ratio (IPR) is calculated yearly. IPR refers to the investment proportion ratio, with regards to the different sizes of rural population among provinces. This is defined in Equation (1):

(1) $$\mathrm{IPR}=\frac{\mathrm{TIPP}}{\mathrm{RPPP}}$$

where TIPP referred to the total investment proportion by province (total investment by province/total investment in the 30 provinces) with regards to improved sanitation; RPPP referred to rural population proportion by province (rural population by province/total rural population in the 30 provinces).

### 2.3. Statistical Analysis

All analyses were further cleaned by using Excel 2007, while the local indicator of spatial auto-correlation (LISA) map which was used to identify spatial clusters and outliers was created using GeoDa 1.8.16 [24]. The Global Moran’s I, local Moran’s I, was calculated by GeoDa, the index and spatial panel models were calculated by R software. All the data was cleaned in the dBase format (DBF) and was connected with China region border shapefile (SHP file extension) via the province ID [4]. The spatial weight was analyzed by the neighboring weights method. The first order queen contiguity was selected as the rule for spatial weights, Hainan province was considered as connected with Guangdong province. 

We use the following methods:

(1) The global spatial autocorrelation method was used to explore the distribution characteristics of ISRs in the provinces [25]. The paper used the Global Moran’s I to analyze the spatial autocorrelation between neighboring regions in the whole 30 provinces: a spatially positive or negative correlation, or spatial independence [26]. The Moran’s I ranges from −0.63783 to 0.96299, the no-spatial autocorrelation value is −0.03448, Moran’s I closer to 0.96299 suggests that the stronger spatial agglomeration, Moran’s I less than −0.03448 indicates a negative autocorrelation. This is defined in Equation (2):
  (2) $$I=\frac{N\sum_{i}\sum_{j}W_{ij}\left(X_{i}-\bar{X}\right)\left(X_{j}-\bar{X}\right)}{\left(\sum_{i}\sum_{j}W_{ij}\right)\sum_{i}{\left(X_{i}-\bar{X}\right)}^{2}}$$
  where *N* was the total number of regions in the study area; *X_i_* and *X_j_* were the ISRs of the regions *i* and *j*, respectively; *W_ij_* was the spatial weight matrix; and $\bar{X}$ was the average of ISRs.
(2) The local spatial autocorrelation was used to explore each region of the distribution. The Moran scatterplot can be divided into four quadrants, representing ISRs of the province and its adjacent provinces: the first is high-high (H–H), which shows a province with a high ISR neighbored by a province also with a high ISR; the second is low-high (L–H), indicating a province with a low ISR adjacent to a province with high ISR; the third is low-low (L–L), showing low ISRs of two adjacent provinces; and the fourth is high-low (H–L), which indicates that a province with a high ISR is neighbored by a province with low ISR. The H–H and L–L are referred to as spatial clusters, while the H–L and L–H are regarded as spatial outliers;
(3) The Theil index was used to analyse the intra-provincial disparities in improved sanitation. The method explores the differences between provincial ISRs. A smaller value of the Theil index represents smaller disparities. Thiel-*T* was used to analyse the provincial disparities. This is defined in Equation (3):
  (3) $$T=\overset{n}{\underset{i=1}{\sum }}\overset{m}{\underset{j=1}{\sum }}\frac{P_{ij}}{P}ln\left(\frac{\frac{P_{ij}}{P}}{\frac{Y_{ij}}{Y}}\right)$$
  where $P_{ij}$ and $Y_{ij}$ were the total number of rural households and the degree of improved sanitation in the *i* region of *j* province, respectively, while *P* and *Y* were the the total number of rural households and the number of improved sanitation in the country, respectively;
(4) Spatial panel model.

Spatial panel data was commonly more informative compared with the traditional models using cross-sectional data. The variables were generally less collinearity, and the models increased efficiency in the estimation [27].

The SLM model was also being named as spatial autoregressive model; it mainly discussed whether the variables had spillover effect in a region. This is defined in Equation (4):

(4) $$y_{it}=\rho \overset{N}{\underset{j=1}{\sum }}W_{ij}y_{jt}+\mu_{i}+x_{it}\beta +\varepsilon_{it}$$

where $y_{it}$ was the dependent variable at spatial unit *i* and time *t*; $X_{it}$ was the observation for the independent variable at *i* and *t*, ρ was the spatial regression coefficient which reflected the spatial dependence of the sample observations, i.e., the direction and degree of influence of the neighboring regions on the observations of the local region, ρ∈[0,1],ρ = 0 indicated the spatial panel model degenerated to a traditional panel model, a high ρ suggested strong spatial autocorrelation, a low value suggested a weak spatial autocorrelation; β was the spatial regression coefficient explaining the relationship between the dependent and independent variables; $\mu_{i}$ represented the spatial specific effects in different spatial units. $\varepsilon_{it}$ was a random error term vector, and was assumed to have a normal distribution; $W_{ij}$ was the spatial adjacent weight matrix, which indicated the spatial adjacent relationship between regions.

## 3. Results

### 3.1. ISR, Standard Deviation and Coefficient of Variation of ISR during the Period 2006–2015

**Figure 1:**
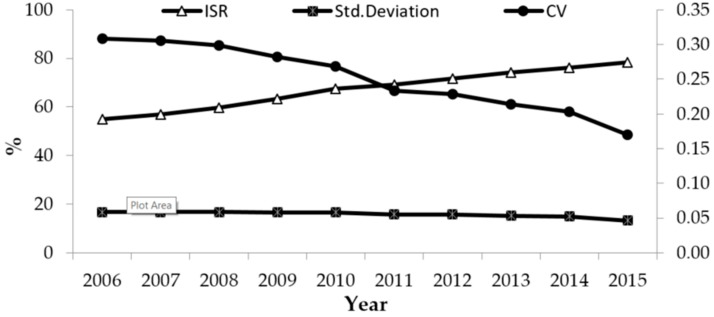
ISR, Standard Deviation and Coefficient of Variation of ISR during the period 2006–2015.

During the period 2006–2015, the ISR increased from 55% to 78.4%, and the standard deviation and CV of ISR decreased from 16.75 to 13.24 and 0.31 to 0.17, respectively (Figure 1). 

China made certain progress in ISR; at the same time, China experienced significant social and economic development over the ten years. The results indicated that both the absolute and relative differences of ISR between provinces decreased during the period 2006–2015. 

**Figure 2:**
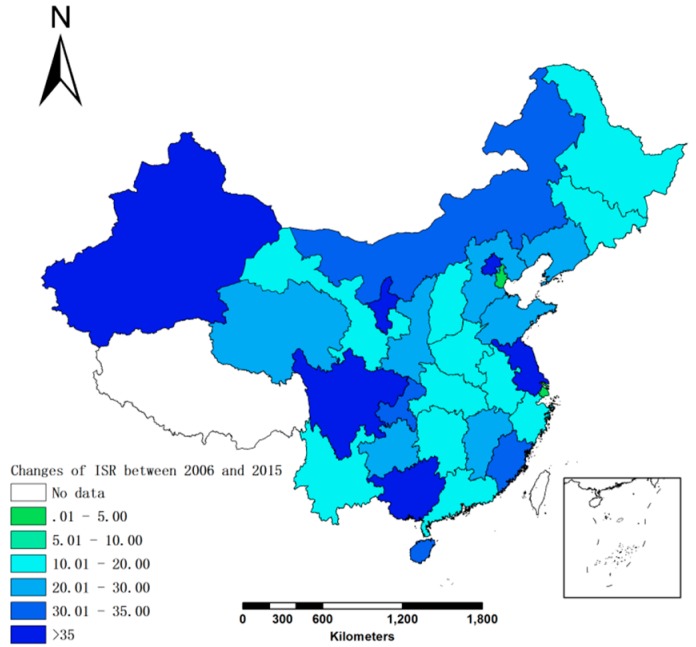
The changes of ISR between 2006 and 2015.

The ISR varied greatly between the provinces. The changes of ISR between 2006 and 2015 were shown in Figure 2. There was a significant difference between the eastern and western parts of the county in 2006, with the western provinces having an ISR of less than 40% and the eastern provinces having an ISR of 65%. This resulted in a difference of 25%, which was reduced to 18.9% in 2015. Compared with 2006, the lowest ISR of 2015 was 54.8%. Furthermore, most provinces had an ISR that was close to 75%, with greater increases in the western provinces (30.9%) compared to the eastern provinces (24.6%). The western provinces with the greater increases in ISR in 2015 were Guangxi (from 43.8% to 85.7%), Sichuan (from 41.7% to 77.7%), Xinjiang (from 36.9% to 75%), and Ningxia (from 36.09% to 70.3%). 

### 3.2. Global Spatial Auto-Correlation

The Moran’s I in 2006–2015, with E (I) as the expected value and *p*-value were shown in Table 2. The Moran’s I values were all with the range of (0.3333, 0.4447), and the Monte Carlo test was significant at the 0.05 level, indicating there was a provincial clustering distribution of ISR in different provinces. 

### 3.3. Local Spatial Auto-Correlation

**Figure 3:**
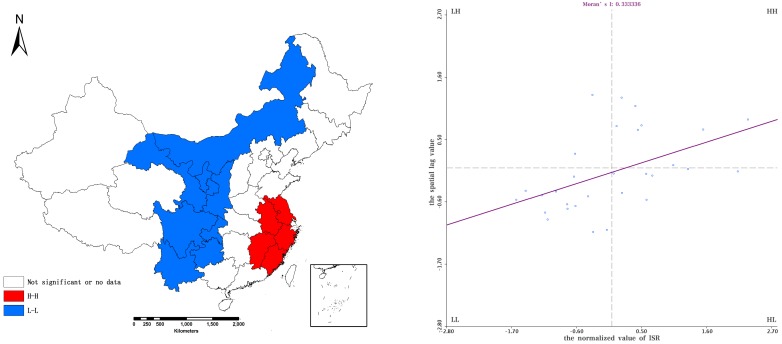
Moran scatter plot of ISR in 2006. The left part shows the corresponding spatial pattern, the right part shows distributions of ISR.

**Figure 4:**
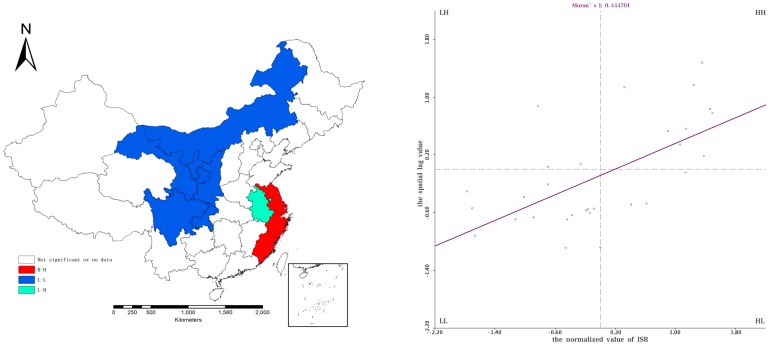
Moran scatter plots of ISR in 2015. The left part shows the corresponding spatial pattern, the right part shows distributions of ISR.

The local spatial auto-correlation distribution of ISR in 2006 and 2015 is shown in Figure 3 and Figure 4, respectively. The H–H and L–L agglomeration provinces experienced little change, with H–H and L–L aggregation located in the Jiangsu-Zhejiang regions and West China, respectively. Most of the provinces had the same ISR distribution in 2006 and 2015, such as the L–L regions of Inner Mongolia, Gansu, Shaanxi and Ningxia during the period 2006–2015. 

Five provinces experienced changes of spatial agglomeration. Yunnan and Guizhou provinces showed L–L aggregation in 2006 but no L–L aggregation in 2015. Jiangxi province showed H–H agglomeration 2006 but no H–H aggregation in 2015. Anhui province showed an H–H aggregation in 2006 as opposed to an L–H aggregation in 2015. Shanxi showed no L–L aggregation in 2006 as opposed to L–L aggregation in 2015. This indicated that Yunnan and Guizhou had a relatively high rate of improvement in sanitation compared to neighboring provinces, while Jiangxi, Anhui and Shanxi provinces had a slower rate of improvement in sanitation compared to neighboring provinces during the period 2006–2015. 

### 3.4. Intra-Provincial Disparities in 2015

Due to the absence of county-level data in Tibet, we used the improved sanitation coverage rates in a final total of 2402 counties among 30 provinces from CHSY 2015 for the disparity analysis.

**Figure 5:**
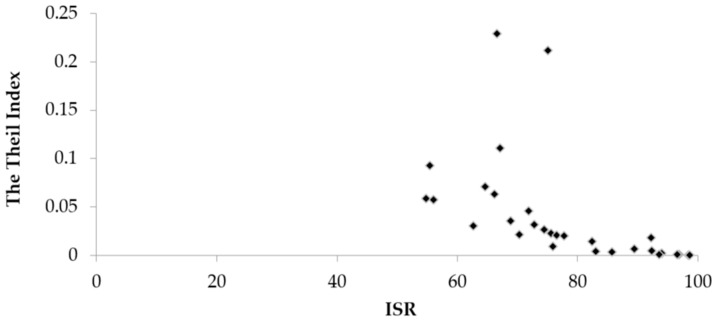
The scatter-plots of the Theil index and ISR among 30 provinces in rural China (2015).

The national Theil index of the improved sanitation in 2015 was 0.05, and the intra-provincial disparities in ISRs were substantial in these provinces. The scatter-plots of the Theil index and the ISRs in rural areas among 30 provinces were shown in Figure 5. 

The correlation analysis of the ISR and the Theil index in the provinces suggested that there was a negative correlation between the ISR and the Theil index (*r* = −0.528, *p* < 0.01). Qinghai (with the highest inequalities), Xinjiang, Anhui, Shaanxi, Yunnan, Chongqing, Guizhou and Shanxi provinces with lower ISRs were more likely to have higher levels of geographical intra-province disparities. In contrast, provinces such as Shanghai (with the lowest inequalities), Beijing, Zhejiang, Tianjin, Jiangsu and Fujian with higher ISRs were more likely to have lower levels of geographical disparities. 

### 3.5. Socio-Economic Factors for ISR

ISR was a dependent variable and 6 independent variables were analyzed from 2006 to 2015. They include socio-economic factors (i.e., GDP per capita, IPR, centralized water supply, rural residents’ expenditure, illiteracy rate of people older than 15, urbanization). We use splm package in the R software program for spatial panel modeling [28]. A spatial panel fixed effects lag model (SLM) was used for analysis of these factors.

The results of the spatial panel model were shown in Table 3. The coefficient of spatial dependence was 0.261, which was significant in the models (*p*-value < 0.001) and suggested the presence of neighborhood effects. The spatial panel modeling analysis suggested that there was a positive association between ISR and GDP per capita (*p*-values < 0.05), IPR (*p*-values < 0.001), centralized water supply (*p*-values < 0.001), rural residents’ expenditure (*p*-values < 0.01), and a negative association between ISR and illiteracy rate of people older than 15 (*p*-values < 0.01). 

These results suggested that by controlling the spatial effect, 1 RMB increase in GDP per capita was related to a 0.00016% (95% CI: 0.000096–0.00023%) increase in ISR; a 1 increase in IPR was associated with a 1.617% (95% CI: 1.339–1.895%) increase in ISR; a one percentage rise in centralized water supply was related to a 0.191% (95% CI: 0.148–0.234%) increase in ISR; a 1 RMB increase in rural residents’ expenditure was associated with a 0.00091% (95% CI: 0.00056–0.00126%) increase in ISR.

There was a negative association between ISR and illiteracy rate of people older than 15 (*p*-values < 0.05), indicated that by controlling the spatial effect, a one percentage increase in illiteracy rate of people older than 15 was associated with a 0.412% (95% CI: 0.257–0.567%) decrease in ISR. 

There was a positive association between ISR and urbanization, but the relationship was not statically significant (*p*-value > 0.05).

## 4. Discussion

In many cases, ensuring an adequate and safe access to water and sanitation is essential to prevent diseases, diminish illness-related poverty and poverty-related illness, which reduce public health expenditure and promote social development [29,30,31]. Latrines seem to only play a small role in our daily lives but could be an important reflection of people’s livelihood. Equal access to clean and sanitation is the fundamental basis of individuals’ prestige [17]. 

This paper explored the spatiotemporal characteristics of ISR. The associations between ISR and socio-economic factors were examined. The results indicated that (1) the provincial improved sanitation rates changes affected both the provinces themselves and the adjacent regions; (2) the H–H and L–L agglomeration provinces experienced little change, with H–H and L–L aggregation located in the Jiangsu–Zhejiang regions and West China, respectively. (3) Provinces with lower improved sanitation rates were more likely to have higher disparities in 2015; the highest and lowest disparities of improved sanitation were located in Qinghai and Shanghai Province, respectively. (4) socio-economic factors such as GDP per capita, IPR, centralized water supply, rural residents’ expenditure was positively affected the ISR, while illiterate rate of people older than 15 was negatively associated with the ISR.

As a whole, the ISRs were increased, and the absolute and relative differences between provinces were decreased during the period 2006–2015. Many countries also made certain progress and variation [32]; some studies suggested that the between-country variation in progress was linked to variations in government policies, institutional commitment and the capacity to execute policies [33]. 

The exploration of the spatiotemporal characteristics of ISR revealed that there were spatial correlations between different provinces; the results suggested that the provincial ISR changes not only affected the provinces, but also affected neighboring areas. Moreover, the local spatial correlation results suggested that the H–H and L–L agglomeration provinces experienced little change, with H–H and L–L aggregations located in the Jiangsu-Zhejiang regions and West China, respectively, in 2006 and 2015. Spatial auto-correlation helped us to pay more attention to L–L aggregation provinces and regions as well as providing insights into the prevention and control of diarrhea, cholera, or other water-borne illnesses [34]. For the five provinces that changed into L–H or L–L aggregations in 2015, the reduction was mainly caused by the increased government investment and higher per-capita income [35] that resulted in an unconscious improvement in latrines [36] and rapid urbanization in neighboring provinces. 

With the addition of intra-provincial disparities in 2015, the provinces with low ISRs were vulnerable areas that are suffering from external environmental changes, such as persistent drought or rainstorm caused by climate change [37], a poor status of deep water tables, emerging environmental pollution, etc. Some researchers suggest that, unless governments and stakeholders deliberately adopted strategies aimed at reaching lowest coverage areas and population groups, it was unlikely that countries will achieve universal coverage [34]. Thus, our results provided targeted support for the proposal of improved sanitation strategies and planning, especially for poor, cold regions, water-deficient counties and minority areas. 

Dialectically speaking, ISR had an intricate relationship with social [38,39,40] and economic factors [41,42,43]. China experienced significant economic development and invested a large amount of funding into the improved sanitation movement, the total investment in sanitation improvement was more than 98 billion (10^9^) Renminbi (RMB) during the period 2006 to 2015, which allowed the Chinese government to build more than 20 million household latrines. In light of the economic development and investment in improved sanitation, we explored and analysed provincial socio-economic panel data (GDP per capita, IPR, centralized water supply, rural residents’ expenditure, illiteracy rate of people older than 15, urbanization) that may affect the ISR by using a spatial panel model. 

The spatial panel analysis results suggested that a 1 RMB increase in GDP per capita was associated with a 0.00016% increase in ISR. GDP per capita was considered to be one of the key socio-economic factors affecting the coverage of ISR [17]. Moreover, we also found that a 1 RMB increase in rural residents’ expenditure was related to a 0.00091% increase in ISR. The two results indicated that improvement in living conditions may increase the ISR. These findings were similar to the findings of previous studies conducted in other regions [44,45,46]. 

We found that IPR was another factor that positively affected ISR. A 1 increase in IPR was related to a 1.617% increase in ISR. IPR reflected the investment proportion with regards to the different sizes of rural population among provinces. Researchers suggested that government-led action was the main driving force for the improvement of rural environmental sanitation in rural China [47]. Residents that lived in undeveloped areas affected by drought were mainly relying on government investments to build facilities for improved sanitation due to their limited sources and poorer hygiene habits [48,49]. 

Our results also indicated that a one percentage rise in centralized water supply was associated with a 0.191% increase in ISR. The results were mostly in consistence with a host of previous researches about the factors associated with improved sanitation usage [50,51]. It is reasonable that good access to external environmental infrastructures such as sufficient clean water, centralized water supply suggests better environmental conditions, more investment, all of which can help increase the coverage rate of improves sanitation [52]. Garn JV [53] and Nakagiri [54] also suggested that better maintenance, accessibility, privacy, facility type, cleanliness, and better hygiene access were all associated with higher improved sanitation coverage. 

We found that a one percentage increase in illiteracy rate of people older than 15 was related to a 0.412% decrease in ISR. The result was similar to the results of some studies which suggested that higher education can raise the awareness of improved sanitation usage and health behavior gradually [55,56]. The results also indicated that ISR in rural areas was constrained not only to income, but also knowledge, altitude and hygiene awareness [57]. 

Although the results of urbanization do not pass the significance test, some researches have suggested that urbanization can help to transmit the modern culture and lifestyle of urban residents to rural residents [58].

For policy makers, in order to increase and equalize ISR, it is first highly recommended to adhere to sanitation improvement led by government, improve the management system and more importantly, strengthen various forms of public health education and promotion [47]. Raising individuals’ awareness of sanitation in areas with relatively low ISRs is the key method to ensure success. The transformation of rural residents’ opinions is a long process that needs the involvement of the whole society, such as government commitment and investments, long-term latrine planning, sustainable promotions and developments, the transformation of scientific and technological achievements as well as health promotion for latrine literacy, which mainly refers to rural residents’ knowledge, altitude and practices of improved sanitation. Moreover, the proper use of improved sanitation can be a more accurate reflection of the access to improved sanitation [59]. Secondly, feasible latrine construction is another practical way to narrow the gap in the improved sanitation rates. Thirdly, the scientific monitoring and effective evaluation of improved sanitation is also an alternative way to close the gap between provinces. 

Our research has some limitations. Firstly, there were maybe some other underlying factors influencing ISR over the years, and a provincial-level spatial scale was used in our study on account of limited data were available for smaller levels (counties or township level). The spatial scale used may obscure some factors via the ecological fallacy effect [60]. Secondly, counties with ISRs equal to zero in two provinces were not involved for the analysis of the Theil index; this may narrow differences between provinces.

## 5. Conclusions

The spatio-temporal characteristics of ISR mainly depend on the comprehensive influence of society, economy, politics and an individual’s way of life. Our results indicated that the provincial improved sanitation rates changes affect both the provinces themselves and the adjacent regions, and the H–H and L–L agglomeration located in the Jiangsu-Zhejiang regions and West China, respectively between 2006 and 2015, socio-economic factors such as GDP per capita, IPR, centralized water supply, rural residents’ expenditure and illiterate rate of people older than 15 affected the ISR. This holds broad implications for policy makers to pay attention not only to infrastructure construction, but also to personal latrine literacy in order to improve ISR. 

## Figures and Tables

**Table 1:** Data sources and expected results (CHSY: China Health Statistical Yearbook; ISR: improved sanitation rate; CSY: China Statistical Yearbook).

Data Sources	Expected Results
CHSY (2006–2015) ISR provinciaL–Level data	Spatial auto-correlation and inter-provincial disparities among 30 provinces during the period 2006–2015;
CHSY (2015) ISR county-level data	Intra-provincial disparities of improved sanitation in 2015;
(1) CHSY (2006–2015) provinciaL–Level data: ISR and Centralized Water Supply rate, total investment of sanitation. (2) CSY (2006–2015) provinciaL–Level data: GDP per capita, rural residents’ expenditure, illiteracy rate of people older than 15, urbanization.	Spatial panel model analysis of 30 provinces during the period 2006–2015.

**Table 2:** Global Moran’s I estimate based on the Monte Carlo test.

Year	Moran’I	Sd.	*p*-Value
2006	0.3333	0.1041	0.0012
2007	0.3532	0.1043	0.0009
2008	0.4097	0.1079	0.0001
2009	0.4361	0.1070	0.0002
2010	0.4215	0.1097	0.0002
2011	0.4166	0.1101	0.0001
2012	0.4217	0.1061	0.0002
2013	0.4330	0.1103	0.0002
2014	0.4272	0.1097	0.0002
2015	0.4447	0.1061	0.0001

Note: the significance test is for marginal values.

**Table 3:** Results of spatial panel model using socio-economic factors.

Variables	Coefficient	S.E.	*t*	*p*
Spatial weight	0.261	0.06	4.35	0.000
GDP per capita (RMB)	0.00016	0.000066	2.452	0.014
IPR	1.617	0.278	5.811	0.000
Centralized water supply (%)	0.191	0.0432	4.421	0.000
rural residents’ expenditure (RMB)	0.00091	0.000348	2.616	0.008
Illiteracy rate of people older than 15 (%)	−0.412	0.155	−2.655	0.008
Urbanization (%)	1.673	1.557	1.075	0.28
